# Assessment of a Model-Informed Precision Dosing Platform Use in Routine Clinical Care for Personalized Busulfan Therapy in the Pediatric Hematopoietic Cell Transplantation (HCT) Population

**DOI:** 10.3389/fphar.2020.00888

**Published:** 2020-07-02

**Authors:** Praveen Shukla, Srijib Goswami, Ron J. Keizer, Beth Apsel Winger, Sandhya Kharbanda, Christopher C. Dvorak, Janel Long-Boyle

**Affiliations:** ^1^ Department of Clinical Pharmacy, University of California, San Francisco, San Francisco, CA, United States; ^2^ Insight Rx, Inc., San Francisco, CA, United States; ^3^ Department of Pediatrics, Division of Hematology and Oncology, University of California, San Francisco, San Francisco, CA, United States; ^4^ Department of Pediatrics, Division of Allergy, Immunology, and Bone Marrow Transplantation, University of California, San Francisco, San Francisco, CA, United States

**Keywords:** busulfan, pharmacokinetics, pediatric, therapeutic drug monitoring, hematopoietic cell transplantation

## Abstract

**Introduction:**

Population pharmacokinetic (PK) studies demonstrate model-based dosing for busulfan that incorporates body size and age improve clinical target attainment as compared to weight-based regimens. Recently, for clinical dosing of busulfan and TDM, our institution transitioned to a cloud-based clinical decision support tool (www.insight-rx.com). The goal of this study was to assess the dose decision tool for the achievement of target exposure of busulfan in children undergoing hematopoietic cell transplantation (HCT).

**Patients and Methods:**

Patients (N = 188) were grouped into cohorts A, B, or C based on the method for initial dose calculation and estimation of AUC: **Cohort A:** Initial doses were based on the conventional dosing algorithm (as outlined in the manufacturers' package insert) and non-compartmental analysis (NCA) estimation using the trapezoidal rule for estimation of AUC following TDM. **Cohort B:** Initial doses for busulfan were estimated by a first-generation PK model and NCA estimation of AUC following TDM. **Cohort C:** Initial doses were calculated by an updated, second-generation PK model available in the dose decision tool with an estimation of AUC following TDM.

**Results:**

The percent of individuals achieving the exposure target at the time of first PK collection was higher in subjects receiving initial doses provided by the model-informed precision dosing platform (cohort C, 75%) versus subjects receiving initial doses based on either of the two other approaches (conventional guidelines/cohort A, 25%; previous population PK model and NCA parameter estimation, cohort B, 50%). Similarly, the percent of subjects achieving the targeted cumulative busulfan exposure (cAUC) in cohort C was 100% vs. 66% and 88% for cohort A and B, respectively. For cAUC, the variability in the spread of target attainment (%CV) was low at 4.1% for cohort C as compared to cohort A (14.8%) and cohort B (17.1%).

**Conclusion:**

Achievement of goal exposure early on in treatment was improved with the updated model for busulfan and the Bayesian platform. Model-informed dosing and TDM utilizing a Bayesian-based platform provides a significant advantage over conventional guidelines for the achievement of goal cAUC exposure.

## Introduction 

Busulfan is a bifunctional alkylating agent commonly used in conditioning regimens prior to hematopoietic cell transplantation (HCT) for the treatment of a variety of childhood diseases, including both malignant and non-malignant disorders. Busulfan has a narrow therapeutic index with improved rates of engraftment and lower drug-related toxicity associated with a cumulative area under the curve (cAUC) of approximately 75–100 mg*h/L (Bolinger et al., 2001; Lindley et al., 2004; Vassal et al., 2008; Bartelink et al., 2016). Due to the erratic population pharmacokinetic (PK) profile in children (Vassal et al., 1989; Slattery et al., 1995; Bolinger et al., 2001; Mccune et al., 2002; Nguyen et al., 2004) and well-described exposure-response relationships, the therapeutic drug monitoring (TDM) for busulfan is performed as a part of the standard clinical care in children undergoing HCT (Vassal et al., 1989; Slattery et al., 1995; Bolinger et al., 2001; Mccune et al., 2002; Nguyen et al., 2004; Palmer et al., 2016).

Historically, wide-spread clinical practice for busulfan dosing has followed the FDA-approved drug label, with an initial busulfan dose estimation based on actual body weight (Anonymous, 2015). This “conventional dosing” nomogram recommends an initial dose of 1.1 mg/kg for patients weighing ≤12 kg and initiating therapy at 0.8 mg/kg/dose for patients weighing >12 kg, regardless of age. Additionally, the European Medicines Agency utilizes a variable dose of busulfan (ranging from 0.8 to 1.2mg/kg) stratified by actual body weight in children <9 kg to >34 kg (Busulfan Fresenius Kabi: EPAR - Product Information). Unfortunately, there are several limitations to these conventional-dose nomograms when applied to clinical use in children, particularly in the very young and this often leads to suboptimal busulfan exposure, graft failure, and significant drug-related morbidity and mortality.

More recently, numerous PK studies have demonstrated population PK models where busulfan clearance (CL) parameter incorporates patient covariates such as body size and age provide improved clinical target attainment when compared to weight-based regimens alone (Bleyzac et al., 2001; Tse et al., 2009; Trame et al., 2011; Bartelink et al., 2012; Paci et al., 2012). Here, we describe our institution's transition to a commercially available, cloud-based clinical decision support decision tool (www.insight-rx.com) for the routine clinical dosing of busulfan and TDM. This easy-to-use Bayesian-based platform helps clinicians individualize busulfan therapy at the point of care using an updated population PK model of busulfan. The primary goal of this study was to assess the performance of the model-informed precision platform for achieving the predefined patient-specific busulfan targeted exposure as compared to older conventional or first-generation model-based strategies.

## Patients and Methods

### Patient Population

All patients and/or guardians provided written informed consent to participate in the routine TDM of busulfan as part of their specific transplant protocol. Consent for participation in the PK analysis was waived as part of the University of California San Francisco Committee on Human Subjects' Research approval process. Eligibility criteria for busulfan PK analysis in this study included (1) subjects between 1 and 26 years of age at the time of HCT; (2) subjects met institutional and protocol specific eligibility criteria for allogeneic HCT that included intravenous busulfan therapy; and (3) patient-specific busulfan plasma time-concentration data were available for analysis. Patients underwent HCT for a wide variety of malignant and non-malignant pediatric disorders. Diagnosis is provided in **Table 1** and demonstrated the inherent differences in the optimal pre-selected busulfan targets between malignant and nonmalignant diseases. In all patients, busulfan was administered intravenously over 2 or 3 h at dose intervals of 6, 12, or 24 h as outlined in the protocol specific combination pre-transplant conditioning regimen. The timing for collection of busulfan PK samples was based on the dose interval and methodology for AUC estimation (trapezoidal rule or the dose decision tool) as previously described (Long-Boyle et al., 2015). Briefly, for every 6- or 12-h dosing, the first PK collection (PK1) occurred with administration of dose 1 or dose 3, followed by repeat assessment with any dose modification. For every 24-h dosing, PK1 was collected following dose 1, with repeat collections occurring with dose 2 and 3, if clinically indicated. Medication with a known, suspected, or theoretical interaction with busulfan based on drug class or shared metabolic pathways were strictly avoided as part of standard of care policies.

**Table 1:** Demographics of patients by cohort (N=188). Data expressed as median (range).

	Cohort A Conventional dosing (N = 53)		Cohort B Model-Based Excel tool (N = 76)		Cohort C Model-Based Bayesian tool (N = 59)	
	Malignant Disease	Non-malignant Disease	Malignant Disease	Non-malignant Disease	Malignant Disease	Non-malignant Disease
**Number of subjects (n)**	25	28	40	36	42	17
**Weight (kg)**	33 (7–101)	18 (3–61)	24 (8–98)	10.1 (4.9–62)	17.8 (8.1–150)	10.2 (5.8–79)
**Age (years)**	8.8 (0.21–29)	4.9 (0.1–21)	6.2 (0.9–24)	1.2 (0.24–19)	5.9 (0.9–20)	1.4 (0.2–17)
**Gender (M/F)**	14/11	17/11	28/12	19/17	28/14	13/4
**Malignant disease**						
Acute myelogenous leukemia	16		17		11	
Chronic myelogenous leukemia	3		3		1	
Juvenile myelomonocytic leukemia	3		3		4	
Myelodysplastic syndrome	3		0		2	
Acute lymphoblastic leukemia	0		9		10	
Neuroblastoma	0		8		10	
Other					4	
**Non-malignant disease**						
Primary immune deficiencies		13		23		9
Inborn errors of metabolism		4		7		3
Hemoglobinopathies		8		6		3
Congenital neutropenia		3		0		2
Predefined cAUC target (mg*h/L)	86 (58–86)	58 (58–86)	82 (62–90)	62 (29–82)	83 (60–90)	60 (17–85)
Observed cAUC target (mg*h/L)	80 (43–110)	72 (53–108)	78 (54–170)	63 (24–102)	83 (59–94)	61 (15–85)

M, male; F, female.

### Study Cohorts

Patient were grouped into cohorts A, B, or C based on the method used for initial dose calculation. Estimation of individual AUC and subsequent dose recommendation were as follows:

**Cohort A:** Initial doses were determined based on the conventional dosing algorithm as outlined in the manufacturers' package insert and non-compartmental analysis (NCA) estimation using trapezoidal rule for estimation of AUC following TDM. Updated individualized doses for days 2–4 were calculated by scaling the previous dose with the ratio of obtained AUC_obs_ and predefined AUC_target_ using the equation and solving for new dose as follows: Dose administered in mg/AUC_obs_ = new dose in mg/desired AUC_target_.
**Cohort B:** Initial doses for busulfan were based on a first-generation PK model as previously described (Savic et al., 2013; Long-Boyle et al., 2015) and NCA estimation using the trapezoidal rule for estimation of AUC following TDM. Individualized doses for days 2–4 were calculated as described for cohort A based on NCA estimates of AUC.
**Cohort C:** Dose estimation by an updated PK model. The refinement of the population PK model is described in the **Supplementary Material**. This updated model incorporates factors of maturation, body size and composition (fat free mass), and conditioning regimen into the PK parameters. Estimation of AUC following TDM was performed using model-informed precision dosing platform. Calculation of doses for days 2–4 were determined by simulation of concentration time course and cumulative exposure from the individualized model. From these simulations, the regimen for the remaining days that would result in cumulative exposure closest to the desired target concentration was identified and recommended.

### Estimation of Cumulative AUC and Target Attainment

For comparing the target attainment between cohorts, the individual cumulative AUC (cAUC) for each patient was derived from the integration of the concentration over time, using the empirical Bayes estimates of individual CL over the entire treatment course as follows:

$$\text{Ratio of AU}{\text{C}}_{\text{obs}}/\text{AU}{\text{C}}_{\mathrm{target}}\;\times 100\%$$

Target attainment was defined as the achieved cAUC divided by the target cAUC for the patient, multiplied by 100%. This calculation was done retrospectively on the collected TDM data for all cohorts, also for those cohorts that were not using model-based dose individualization (A and B). Given time limitations required for bioanalytical assessment of plasma concentrations by mass spectrometry, PK assessments for the majority of subjects was performed on only the first 3 of 4 days of therapy. Only for a few patients sampling was performed on day 4 as well. Thus, for most patient's exposure on the 4^th^ day of therapy was extrapolated using the individual PK parameter estimates determined from TDM performed with the first 3 days of treatment.

### Statistical Analysis

To assess the performance of the model-informed precision dosing software as compared to other cohorts the ratio of individual AUC_obs_ to the predefined individual AUC_target_ was calculated for both PK1 AUC_obs_/AUC_target_ and the entire course of therapy defined by cAUC_obs_/cAUC_target_. One-way analysis of variance with *post hoc* Bonferroni adjustment for multiple comparisons was used to compare differences in PK1 among the cohorts. Results are expressed as median (range) and a p-value of <0.05 was considered to be statistically significant. Finally, the percentage of subjects achieving 80–120%, less than 80%, or greater than 120% of the predefined busulfan target at the time PK1 assessment were calculated and presented.

## Results

**Table 1** shows the baseline characteristics of the pediatric HCT patient cohorts who received busulfan as a part of conditioning regimen (N = 188). The busulfan dose, dosing interval, and cAUC target varied among patient cohorts based on several factors including changes in standard-of-care practices for administration of busulfan with time, indication for transplant, and patient-specific comorbidities at the time of transplant.

**Figure 1:**
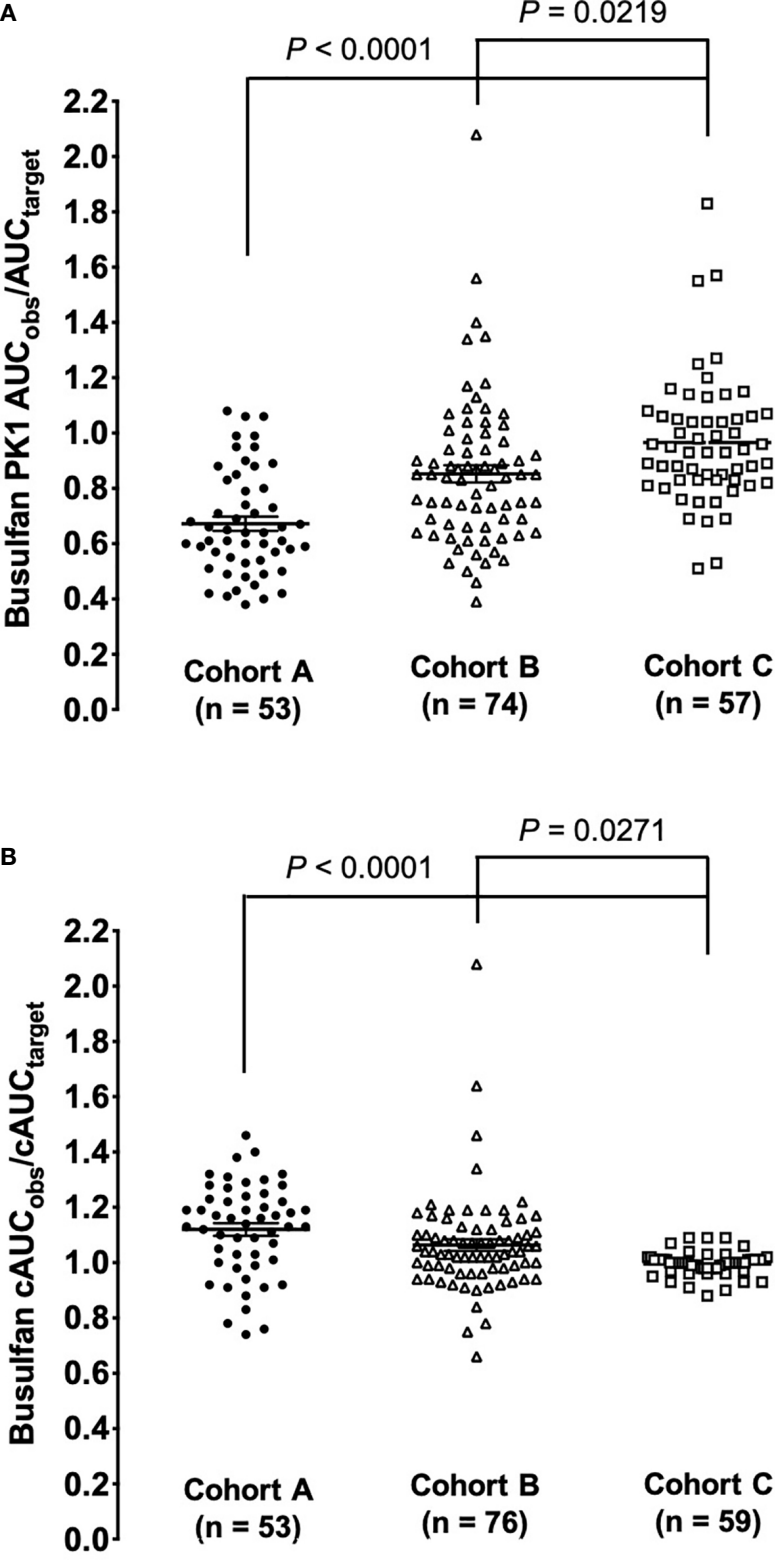
Comparison of the ratio of observed AUC verses goal exposure for the three cohorts. **(A)** The ratio of busulfan 1st dose PK observed to the pre-defined AUC target. **(B)** The ratio of busulfan cAUC observed to the pre-defined cAUC target.

The estimated ratio of PK1 AUC_obs_/AUC_target_ and cAUC_obs_/cAUC_target_ are shown in **Table 2**. For the PK1 assessment the median (range) ratio of the AUC_obs_/AUC_target_ for cohort C was closest to one at 0.93 (0.51–1.8), followed second by cohort B [0.85 (0.39–2.1)] and lastly cohort A [0.64 (0.38–1.10)]. A similar trend for the ratio closest to one was also shown with cohort C for cAUC_obs_/cAUC_target_ when dose estimation was performed by the dose decision tool as compared to cohort A or B. The improvement shown in cohort C in achieving the predefined target exposure was statistically significant as compared to cohorts A and B (**Figures 1A, B**).

**Table 2:** Comparison of the ratio of observed verses pre-defined goal AUC for 1st PK assessment and overall cAUC.

	Cohort A	Cohort B	Cohort C
**1^st^ PK Assessment**			
Ratio of AUC_obs_/AUC_target_ Median (range)	0.64 (0.38–1.1)	0.85 (0.39–2.1)	0.93 (0.51–1.8)
Coefficient of variation (%CV)	28%	31%	24%
Number of subjects	53	74	57
**Overall cAUC**			
Ratio cAUC_obs_/cAUC_target_ Median (range)	1.10 (0.74–1.5)	1.00 (0.66–2.1)	1.00 (0.88–1.10)
Coefficient of variation (%CV)	15%	17%	4%
Number of subjects	53	76	59

Data expressed as median (range). AUC_obs_, area under the curve observed; AUC_target_, area under the curve target; cAUC_obs_, cumulative area under the curve observed; cAUC_target_, cumulative area under the curve target; CV, coefficient of variation.

**Tables 3** and **4** provide the percent of subjects falling within 80–120% of the predefined exposure target for the first dose and desired cAUC. Individuals achieving the predefined AUC_target_ for day 1 were higher in subjects receiving initial doses based on the updated model-informed precision dosing tool (cohort C, 75%) versus conventional guidelines or older PK model at 25% and 50% for cohort A and B, respectively. Similarly, the percent of subjects achieving the cAUC_target_ was highest in cohort C (100%). For individuals with a busulfan cAUC_obs_ outside the predefined therapeutic range, the PK1 exposure was more often sub-therapeutic. Subtherapeutic 1^st^ dose PK assessments AUC_obs_ (defined as % of subjects with <80% of the predefined 1^st^ dose AUC target) occurred in 75% of patients in cohort A and 43% for cohort B, as compared to only 16% in cohort C.

**Table 3:** Comparision of percent of subjects achieving the pre-defined busulfan exposure (AUC) for the 1st PK assessment.

	Number (%) of subjects within 80–120% of the predefined 1^st^ dose AUC target	Number (%) of subjects with <80% of the predefined 1^st^ dose AUC target	Number (%) of subjects with >120% of the predefined 1^st^ dose AUC target
**Cohort A** **(N = 53)**	13 (25%)	40 (75%)	0 (0%)
**Cohort B** **(N = 74)**	37 (50%)	32 (43%)	5 (7%)
**Cohort C** **(N = 57)**	43 (75%)	9 (16%)	5 (9%)

Data expressed as number of subjects (%).

**Table 4:** Comparison of the percent of subjects achieving the targeted cumulative busulfan exposure (cAUC) for three different dosing cohorts.

	Number (%) of subjects within 80–120% of the predefined cAUC target	Number (%) of subjects with <80% of the predefined cAUC target	Number (%) of subjects with >120% of the predefined cAUC target
**Cohort A** **(N = 53)**	35 (66%)	3 (6%)	15 (28%)
**Cohort B** **(N = 76)**	67 (88%)	3 (4%)	6 (8%)
**Cohort C** **(N = 59)**	59 (100%)	0 (0%)	0 (0%)

Data expressed as Data expressed as number of subjects (%).

## Discussion

This work is the first to compare different strategies for determining initial doses of busulfan in combination with TDM for goal target exposure attainment. We found that combining model-informed precision dosing and TDM significantly improves the busulfan cAUC_obs_/cAUC_target_ in pediatric HCT patients, as compared to conventional weight-based methods. This improvement in busulfan exposure increases our ability to achieve the predefined individual therapeutic window early on in the treatment course, which is expected to improve clinical outcomes. Our analysis also demonstrates the importance of continuous learning with improved therapeutic target attainment through a process that incorporates model evaluation and re-estimation of model parameters (Keizer et al., 2018) with additional patient data. Finally, these data reinforce the clinical utility for a model-informed precision dosing platform to support personalized busulfan dosing and TDM over historical methods for achievement of cAUC_target_.

It is well-recognized that the PK and exposure-response relationships of drugs can differ widely between children and adults (van Den Anker et al., 2011). Particularly within the first year of life, age-related differences in both physiologic and metabolic processes can significantly alter PK and drug disposition (Hines, 2008). Relationships between a dose of drug administered, circulating drug concentrations, and pharmacodynamic endpoints may vary across different age groups and disease states. This has been shown to be true for busulfan, a critical component of pretransplant conditioning in HCT. It has been well described that achieving a target busulfan exposure is necessary to support a successful HCT in children. Sub-therapeutic busulfan levels are associated with graft failure (Mccune et al., 2000; Lindley et al., 2004; Nguyen et al., 2004), while supra-therapeutic exposure can lead to severe drug-related toxicity, including mucositis and sinusoidal obstruction syndrome (Slattery et al., 1995; Bolinger et al., 2001). Therefore, TDM, performed through the collection of serial blood samples, is necessary to optimize systemic exposure. In the setting of pediatric HCT, cAUC of 75–100 mg*h/L over 4 days of therapy has been shown to increase the likelihood of event-free survival for a variety of both malignant and non-malignant disorders (Bolinger et al., 2001; Vassal et al., 2008; Bartelink et al., 2016). However, optimal exposure may differ from this range for an individual based on several factors, including heavy pretreatment with high-dose chemotherapy, comorbidities, diagnosis (malignant vs. nonmalignant), and other myeloablative agents included in pre-transplant combination chemotherapy (Mccune et al., 2000; Bolinger et al., 2001; Law et al., 2012). Similarly, the dose interval and total number of days of therapy is highly variable among the different clinical sites and disease-specific protocols. Conventional dosing for busulfan as recommended by the manufacturer cannot account for such differences, often leading to suboptimal exposure (Savic et al., 2013; Long-Boyle et al., 2015). In this study, we found only a quarter of patients who receive conventional dosing (cohort A) achieved the predefined goal exposure for busulfan after the first dose, with 75% of subjects achieving subtherapeutic exposure. This level of exposure is not acceptable, as 75% of patients are under-dosed, and thus at risk of graft failure and other complications. We show that model-informed precision dosing provides a significant advantage over the conventional guidelines, improving individualized therapy irrespective of the therapeutic target or dosing interval.

**Table 1** displays the difference in the median weight from cohort A to cohorts B/C. This difference is most likely attributed to changes in the type and age of children able to undergo HCT for the correction of their disease. More recently, several advancements in HCT related to diagnosis and dosing of busulfan have enabled us to transplant more children at a much younger age. Particularly, the implementation of newborn screening for early diagnosis (within the first 7 days of life) of primary immune deficiencies and inborn error of metabolism. Thanks to newborn screening children can now be transplanted at <6 months of life. Previously, these children, if they survived infancy, would be diagnosed at an older age when disease symptoms were finally recognized. By developing and using population PK models at the bedside, we have been able to facilitate safer transplants through better dosing of busulfan for very young children.

The effect of this difference between the groups should have very little impact on the results, provided weight and age are included in the final covariate model that is used to estimate individual CL and AUC (**Supplementary Material**).

Given the short duration of busulfan therapy (2 to 4 days), achievement of target exposure early on in treatment, preferably with the first dose, is crucial. Most centers do not have the ability to measure busulfan levels through an “on site” clinical laboratory, limiting the ability to perform early dose modifications. As shown in this analysis, a clinical decision support platform that can enable model-informed precision dosing can generate initial doses for busulfan that improves the chance of achieving therapeutic targets with the first dose. Early target attainment directly leads to reduction in the number of PK assessments with additional doses, enhancing patient safety by minimizing blood loss and decreasing the need for repeated accessing of indwelling IV catheters. This is in comparison to traditional PK methodologies, such as non-compartmental analyses, which requires a substantial number of blood collections to accurately estimate PK parameters. In contrast, the model-informed precision dosing platform supports the use of more innovative blood collection strategies, such as D-optimality-based or sparse sampling, to limit blood collections. This enhances patient safety by reducing turn-around time for reporting PK results from external labs, allowing for dose modifications earlier in therapy and overall requiring fewer blood collections.

Our institution began using (preliminary versions of) the model-informed precision dosing tool for busulfan approximately 5 years ago. This has allowed clinicians to quickly determine a patient-specific initial busulfan dose based on the child's age and weight. Clinicians also determine dose modifications based on TDM irrespective of the dosing interval or goal exposure. The implementation of this dosing platform has been very successful, and clinicians have found it easy to learn and use. One primary advantage of this particular platform was that, in addition to individualized dosing, the population PK model could be rapidly updated and refined as new data was entered into the system, further improving model accuracy and precision. Using this approach, the model estimates were updated to better describe busulfan exposure in both neonates and children undergoing HCT, resulting in a significantly improved fit (cohort C) as compared to the first-generation population PK model supported by the software (cohort B). The current analysis demonstrates the importance of continuous learning through external model validation and the re-estimation process of PK parameters (Keizer et al., 2018) to improve dosing in patients with a wide range of weights and ages. Specifically, this has allowed for safe implementation into novel clinical trials utilizing busulfan as part of HCT conditioning regimens such as “low-exposure” busulfan (Dvorak et al., 2019) and gene therapy for infants less than 6 months of life diagnosed with severe combined immunodeficiencies (Mamcarz et al., 2019).

Although the implementation of the model-informed precision dosing platform significantly improved clinical target attainment for patients receiving busulfan, limitations remain. Even with the application of model-informed precision dosing, especially given the fact that busulfan shows considerable between-day variability, there will be a proportion of patients who will fail to achieve optimal exposure with dose 1, thus necessitating repeat blood collections and dose modifications. No model is perfect and continuous learning with model updates within the applied patient population is still required. Additional clinical or patient-specific covariates which are not accounted for in the current model may be important determinants of busulfan CL. These include specific disease or diagnoses, genetic variants in drug metabolizing enzymes involved in busulfan metabolism, and drug interactions including enzyme inducers or inhibitors (Vassal et al., 1993; Bertholle-Bonnet et al., 2007; Johnson et al., 2008; Zwaveling et al., 2008; Ansari and Krajinovic, 2009; Abbasi et al., 2011). At our institution, we take a very active approach to avoid concomitant administration of medications that are known, suspected, or may theoretically interact with busulfan based on drug class or shared metabolic pathways. This level of scrutiny for potential drug interactions may not be similar among different transplant centers.

In summary, this work reinforces the clinical utility for a Bayesian-based, model-informed precision platform and TDM of busulfan over historical methods for achievement of cAUC_target_. Continuous learning through model evaluation and re-estimation of PK parameters with additional data is critical to further improve precision dosing in applied populations with population-derived models.

## Data Availability Statement

The control stream for the final PK model has been provided in the **Supplemental Material**. The PK data may be provided by contacting the corresponding author and approval by the UCSF IRB.

## Ethics Statement

The studies involving human participants were reviewed and approved by UCSF IRB Committee. Written informed consent to participate in this study was provided by the participants' legal guardian/next of kin.

## Author Contributions

PS, SG, RK, BW, SK, CD, and JL-B designed the study, enrolled patients, searched the published work, analyzed and interpreted data, wrote the report, reviewed the manuscript, and approved the final draft.

## Funding

This study was funded by an anonymous donor (JL-B).

## Conflict of Interest

SG and RK are cofounders of and have equity in InsightRx. JL-B currently serves as a scientific consultant to InsightRX.

The remaining authors declare that the research was conducted in the absence of any commercial or financial relationships that could be construed as a potential conflict of interest.

## References

[B1] Abbasi N. Vadnais B. Knutson J. A. Blough D. K. Kelly E. J. O'donnell P. V. (2011). Pharmacogenetics of intravenous and oral busulfan in hematopoietic cell transplant recipients. J. Clin. Pharmacoly. 51, 1429–1438. 10.1177/0091270010382915 PMC3117932 21135089

[B2] Anonymous (2015). Product Information: Busulflex (busulfan) For Injection. Toyko, Japan: Otsuka Pharmaceuticals Co.

[B3] Ansari M. Krajinovic M. (2009). Can the pharmacogenetics of GST gene polymorphisms predict the dose of busulfan in pediatric hematopoietic stem cell transplantation? Pharmacogenomics 10, 1729–1732. 10.2217/pgs.09.135 19891548

[B4] Bartelink I. H. Van Kesteren C. Boelens J. J. Egberts T. C. Bierings M. B. Cuvelier G. D. (2012). Predictive performance of a busulfan pharmacokinetic model in children and young adults. Ther. Drug Monit. 34, 574–583. 10.1097/FTD.0b013e31826051bb 22972539

[B5] Bartelink I. H. Lalmohamed A. Van Reij E. M. Dvorak C. C. Savic R. M. Zwaveling J. (2016). Association of busulfan exposure with survival and toxicity after haemopoietic cell transplantation in children and young adults: a multicentre, retrospective cohort analysis. Lancet Haematol. 3, e526–e536. 10.1016/S2352-3026(16)30114-4 27746112 PMC5159247

[B6] Bertholle-Bonnet V. Bleyzac N. Galambrun C. Mialou V. Bertrand Y. Souillet G. (2007). Influence of underlying disease on busulfan disposition in pediatric bone marrow transplant recipients: a nonparametric population pharmacokinetic study. Ther. Drug Monit. 29, 177–184. 10.1097/FTD.0b013e318039b478 17417071

[B7] Bleyzac N. Souillet G. Magron P. Janoly A. Martin P. Bertrand Y. (2001). Improved clinical outcome of paediatric bone marrow recipients using a test dose and Bayesian pharmacokinetic individualization of busulfan dosage regimens. Bone Marrow Transplant. 28, 743–751. 10.1038/sj.bmt.1703207 11781625

[B8] Bolinger A. Zangwill A. Slattery J. Risler L. Sultan D. Glidden D. (2001). Target dose adjustment of busulfan in pediatric patients undergoing bone marrow transplantation. Bone Marrow Transplant. 28, 1013–1018. 10.1038/sj.bmt.1703264 11781609

[B9] Dvorak C. C. Long-Boyle J. Dara J. Melton A. Shimano K. A. Huang J. N. (2019). Low Exposure Busulfan Conditioning to Achieve Sufficient Multi-lineage Chimerism in Patients with Severe Combined Immunodeficiency. Biol. Blood Marrow Transplant. 25 (7), 1355–1362. 10.1016/j.bbmt.2019.03.008 30876930 PMC7384257

[B10] Hines R. N. (2008). The ontogeny of drug metabolism enzymes and implications for adverse drug events. Pharmacol. Ther. 118, 250–267. 10.1016/j.pharmthera.2008.02.005 18406467

[B11] Johnson L. A. Orchard P. J. Baker K. S. Brundage R. Cao Q. Wang X. (2008). Glutathione S-transferase A1 genetic variants reduce busulfan clearance in children undergoing hematopoietic cell transplantation. J. Clin. Pharmacol. 48, 1052–1062. 10.1177/0091270008321940 18635758 PMC3204946

[B12] Keizer R. J. Ter Heine R. Frymoyer A. Lesko L. J. Mangat R. Goswami S. (2018). Model-Informed Precision Dosing at the Bedside: Scientific Challenges and Opportunities. CPT Pharmacometrics Syst. Pharmacol. 7, 785–787. 10.1002/psp4.12353 30255663 PMC6310898

[B13] Law J. Cowan M. J. Dvorak C. C. Musick L. Long-Boyle J. R. Baxter-Lowe L. A. (2012). Busulfan, fludarabine, and alemtuzumab as a reduced toxicity regimen for children with malignant and nonmalignant diseases improves engraftment and graft-versus-host disease without delaying immune reconstitution. Biol. Blood Marrow Transplant. 18, 1656–1663. 10.1016/j.bbmt.2012.05.006 22609040

[B14] Lindley C. Shea T. Mccune J. Shord S. Decker J. Harvey D. (2004). Intraindividual variability in busulfan pharmacokinetics in patients undergoing a bone marrow transplant: assessment of a test dose and first dose strategy. Anti-cancer Drugs 15, 453–459. 10.1097/01.cad.0000127145.50172.51 15166618

[B15] Long-Boyle J. Savic R. Yan S. Bartelink I. Musick L. French D. (2015). Population pharmacokinetics of busulfan in pediatric and young adult patients undergoing hematopoietic cell transplant: a model-based dosing algorithm for personalized therapy and implementation into routine clinical use. Ther. Drug Monit. 37, 236. 10.1097/FTD.0000000000000131 25162216 PMC4342323

[B16] Mamcarz E. Zhou S. Lockey T. Abdelsamed H. Cross S. J. Kang G. (2019). Lentiviral gene therapy combined with low-dose busulfan in infants with SCID-X1. N. Engl. J. Med. 380, 1525–1534. 10.1056/NEJMoa1815408 30995372 PMC6636624

[B17] Mccune J. S. Gibbs J. P. Slattery J. T. (2000). Plasma concentration monitoring of busulfan. Clin. Pharmacokinet. 39, 155–165. 10.2165/00003088-200039020-00005 10976660

[B18] Mccune J. Gooley T. Gibbs J. Sanders J. Petersdorf E. Appelbaum F. (2002). Busulfan concentration and graft rejection in pediatric patients undergoing hematopoietic stem cell transplantation. Bone Marrow Transplant. 30, 167–173. 10.1038/sj.bmt.1703612 12189535

[B19] Nguyen L. Fuller D. Lennon S. Leger F. Puozzo C. (2004). IV busulfan in pediatrics: a novel dosing to improve safety/efficacy for hematopoietic progenitor cell transplantation recipients. Bone Marrow Transplant. 33, 979–989. 10.1038/sj.bmt.1704446 15064687

[B20] Paci A. Vassal G. Moshous D. Dalle J.-H. Bleyzac N. Neven B. (2012). Pharmacokinetic behavior and appraisal of intravenous busulfan dosing in infants and older children: the results of a population pharmacokinetic study from a large pediatric cohort undergoing hematopoietic stem-cell transplantation. Ther. Drug Monit. 34, 198–208. 10.1097/FTD.0b013e31824c2f60 22406655

[B21] Palmer J. McCune J. S. Perales M. A. Marks D. Bubalo J. Mohty M. (2016). Personalizing Busulfan-Based Conditioning: Considerations from the American Society for Blood and Marrow Transplantation Practice Guidelines Committee. Biol. Blood Marrow Transplant. 22, 1915–1925. 10.1016/j.bbmt.2016.07.013 27481448

[B22] Savic R. M. Cowan M. J. Dvorak C. C. Pai S. Y. Pereira L. Bartelink I. H. (2013). Effect of weight and maturation on busulfan clearance in infants and small children undergoing hematopoietic cell transplantation. Biol. Blood Marrow Transplant. 19, 1608–1614. 10.1016/j.bbmt.2013.08.014 24029650 PMC3848313

[B23] Slattery J. T. Sanders J. E. Buckner C. D. (1995). Graft-rejection and toxicity following bone marrow transplantation in relation to busulfan pharmacokinetics. Bone Marrow Transplant. 16 (1), 31–42. 7581127

[B24] Trame M. N. Bergstrand M. Karlsson M. O. Boos J. Hempel G. (2011). Population pharmacokinetics of busulfan in children: increased evidence for body surface area and allometric body weight dosing of busulfan in children. Clin. Cancer Res. 17, 6867–6877. 10.1158/1078-0432.CCR-11-0074 21918171

[B25] Tse W. Duerst R. Schneiderman J. Chaudhury S. Jacobsohn D. Kletzel M. (2009). Age-dependent pharmacokinetic profile of single daily dose iv busulfan in children undergoing reduced-intensity conditioning stem cell transplant. Bone Marrow Transplant. 44, 145–156. 10.1038/bmt.2008.437 19182832

[B26] van den Anker J. N. Schwab M. Kearns G. L. (2011). Developmental pharmacokinetics.Handb Exp Pharmacol. 205, 51–75. 10.1007/978-3-642-20195-0_2 21882105

[B27] Vassal G. Gouyette A. Hartmann O. Pico J. Lemerle J. (1989). Pharmacokinetics of high-dose busulfan in children. Cancer Chemother. Pharmacol. 24, 386–390. 10.1007/BF00257448 2791192

[B28] Vassal G. Fischer A. Challine D. Boland I. Ledheist F. Lemerle S. (1993). Busulfan disposition below the age of three: alteration in children with lysosomal storage disease. Blood 82, 1030–1034. 10.1182/blood.V82.3.1030.1030 8338934

[B29] Vassal G. Michel G. Esperou H. Gentet J. Valteau-Couanet D. Doz F. (2008). Prospective validation of a novel IV busulfan fixed dosing for paediatric patients to improve therapeutic AUC targeting without drug monitoring. Cancer Chemother. Pharmacol. 61, 113–123. 10.1007/s00280-007-0455-2 17393167

[B30] Zwaveling J. Press R. R. Bredius R. G. Van Derstraaten T. R. Den Hartigh J. Bartelink I. H. (2008). Glutathione S-transferase polymorphisms are not associated with population pharmacokinetic parameters of busulfan in pediatric patients. Ther. Drug Monit. 30, 504–510. 10.1097/FTD.0b013e3181817428 18641537

