# Supplementary material for: Assessment of a Model-Informed Precision Dosing Platform Use in Routine Clinical Care for Personalized Busulfan Therapy in the Pediatric Hematopoietic Cell Transplantation (HCT) Population
Source: Front Pharmacol. 2020 Jul 2;11:888. doi: 10.3389/fphar.2020.00888 (PMC7351521; doi:10.3389/fphar.2020.00888)
Supplement: Supplementary file 1 [file DataSheet_1.docx]

# Supplemental Materials: Busulfan Population PK Model Development

# Introduction

This appendix describes a modeling analysis aimed at updating the population PK model used for pediatric dosing of busulfan in the InsightRX platform. The original models were described in Savic et al. / Long-Boyle et al. [1,2]) and were re-evaluated and revised based on newly collected TDM data from routine care of patients at UCSF. The main reason for updating the models was a concern that, due to the introduction of a new BMT conditioning regimen (Bu/Flu/Clo), some patients were dosed too high initially. Additionally, we aimed to develop a joint model for the entire age and weight range in pediatric patients.

# Methods

## Data

Data from the studies described above [1,2] were available and used in this study as well. Details regarding data collection, analytical methods, and ethical approval are found in the respective articles.

Newly collected plasma samples were analyzed by the University of California San Francisco, Department of Pathology and Laboratory Medicine using a validated liquid chromatography with tandem mass spectrometry assay described elsewhere in detail. The assay was linear in the range of 6–2,000 ng/mL. Assay intraday and interday precision were <3% and <6%, respectively. Local Institutional Review Boards approved this study, and written informed consent to undergo therapy and PK studies was obtained from all patients and guardians.

In total 3,580 samples from 299 patients were available for analysis.

## Data cleaning

In total, 15 TDM concentrations (0.4%) were removed from the joint datasets (including from the newly collected data) due to an unexplainable deviation from expected concentration. Most commonly, trough observations were labeled as taken after the start of the next dose. Since it was not possible to tease out whether the observation was actually taken after start of the new dose, or were recorded in error, they were removed from the dataset. Additonally, it was observed in both datasets that a fraction (5 samples in total) of the observations taken directly after end of infusion were much higher than expected based upon the model (e.g. 6,000 vs 3,000 ng/mL). Fitting a 2-compartment model structure did not improve the description of these data points, and inclusion of these data biased the individual estimates of CL / V for these individuals in the 1-compartment setting. Therefore these points were removed from the dataset.

## Model development

Re-evaluation of model structure and parameter estimates was initially performed separately for the two referenced models, which were built on data of patients >12 kg (Long-Boyle et al.) and <12 kg (Savic et al.). For both PK models the most important structural model characteristics (number of compartments, non-linearities, covariates etc.) were re-evaluated using a combined dataset of the original and newly collected data.

# Results

## Model: weight >12kg

- *elimination*: a non-linear CL (like included in the original model Long-Boyle et al.) could not be identified from the current data: fit was not significantly better with a Michaelis-Menten PK model, and the KM value increased to infinity when estimation of this parameter was attempted.
- *distribution*: No improvement in fit was observed with an added peripheral compartment.
- *covariates*:
  - **CL~age**: A hinge function for CL~AGE as used in the original model indeed proved significant and relevant improvement in fit over no relationship with age. However, the slope for weights higher than the hinge-point was not significantly different from 0. Also, plots of the AGE vs $\eta_{1}$ (when AGE was not included in the model), showed that the optimal hinge point was probably much lower than 12 yrs as used in the original model (see figure below). The optimal hinge point could not reliably be estimated, so this was initially fixed at 3 years. However, a maturation function for age (as included in the original model for <12kg) proved to show a much better fit than a hinge function. The half-life and maximal effect of the maturation function were initially estimated. However, since the estimated values were very close to those estimated from the <12kg dataset, the estimates were fixed to those (1.44 yrs), since that dataset contained denser information in the relevant age region.
  - **CL~weight**: Weight was retained in the model as it was a very significant predictor, even after accounting for age. However, instead of *total body weight*, allometric scaling was also attempted using FFM, with FFM estimated based on age, sex, weight and height using the equation from Al-Sallami et al. for children.[3] This provided significantly better fit, and reduced the correlation that was observed in the plot of $\eta_{1}$~WT. Scaling by a weighted average of FFM and weight (as was reported by McCune et al. [4]) provided no benefit to scaling by FFM alone.
  - **CL~day**: CL on day one was significantly lower (15%) than on the other days. This effect was also observed in Bartelink et al.[5] but not reported in McCune et al (although it is unclear whether this was evaluated in the latter analysis).
  - **V~weight**: the significant relationship was confirmed and therefore retained in the model. Similar to CL, this was converted to an allometric implementation based on FFM instead of total body weight.


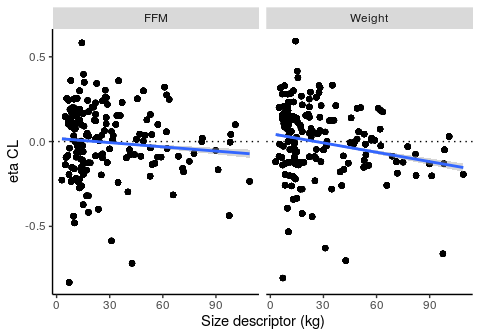


Remaining variability in CL (eta1) versus weight, when CL and V scaled by weight


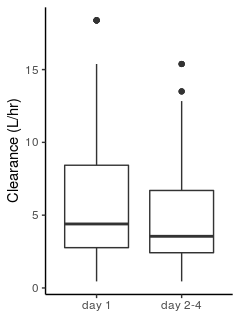


Busulfan clearance versus treatment day

- *random effects*: The variability components described in the original article were still siginficant and clinically relevant. The only addition that was made was the inclusion of inter-occasion variability on volume of distribution, which significantly improved model fit and reduced residual error.


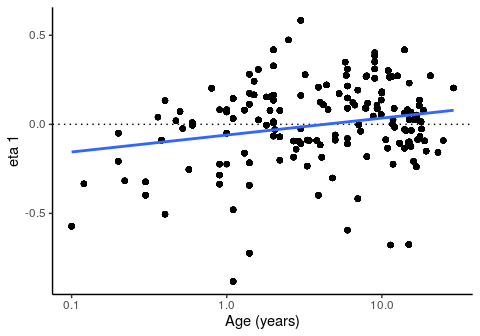


Remaining variability in CL (eta1) versus age


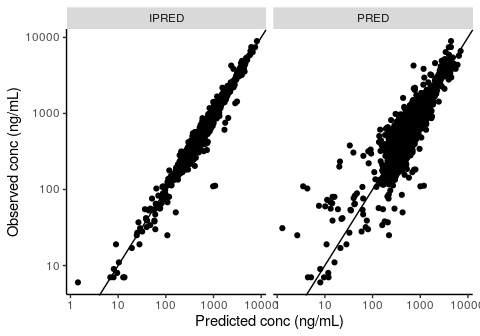

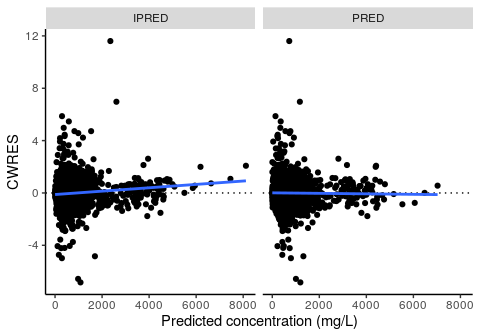


When the *conditioning regimen type* was included as a binary covariate on CL, it improved fit considerably and significantly, and CL was estimated to be ~19% lower on average for the patients on the Bu/Flu/Clo conditioning regimen. The visual predictive check (not included here) showed no apparent bias in goodness of fit plots over the entire timecourse.

## Model: weight <12kg

- *elimination*: similar to the original model, a non-linear CL could not be identified from the data: fit was not significantly better with a Michaelis-Menten PK model, and the KM value increased to infinity when attempted to estimate it.
- *distribution*: addition of a peripheral compartment provided significantly better fit ($p$ < 0.01). However, the model parameters for peripheral distribution (Q and V2) where estimated at physiologically unrealistic values, five orders of magnitude higher than CL and V. This indicated that the peripheral compartment modeled as such primarily described the initial 15 minutes after infusion where the drug concentration spiked, perhaps due to the non-complete drug distribution, similar to what was reported by McCune et al, but not observed by Bartelink et al. After removal of the most extreme datapoints that showed this phenomenon (n=6), addition of the peripheral compartment was not significant. As in our view the peripheral compartment only attempts to describe incomplete distribution over the body, and the phenomenon was only observed in a low number of patients, it was chosen not to include the peripheral compartment in the final model.
- *covariates*:
  - **CL~age**: a hinge function (as used in model >12kg) showed worse fit than the 2-parameter maturation function. As in the original model, both parameters of the maturation function could be estimated with good precision.
  - **CL~weight**: the relationship was confirmed to be significant, and retained in model but scaled now allometrically by FFM, as described above for the >12 kg model.
  - **CL~day**: this relationship was included in model, as described above.
  - **V~weight**: confirmed significant and retained in model. Also scaled allometrically by FFM instead of WT.
- *random effects*: Inter-occasion variability was included also on Volume of distribution, which significantly improved model fit and reduced residual error. For the residual error model, the original PK model contained three separate error magnitudes for data from three different centers. For the current data, the proportional errors for all three centers were estimated at around 9%, while only the additive part varied 15-53 ng/mL between the centers. While the inclusion of separate error magnitude did indeed improve the model fit for these data, it does not render the popPK model more predictive for future patients and poses a problem for use of the model in new populations. It was therefore chosen to estimate a joint residual error magnitude for the proportional and additive errors for data from all centers.


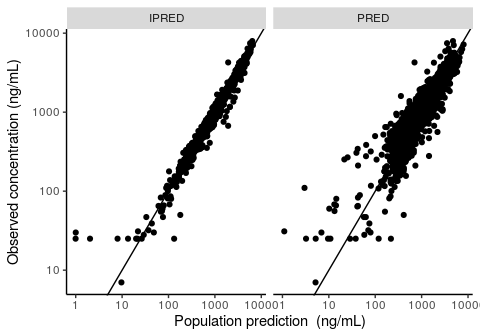

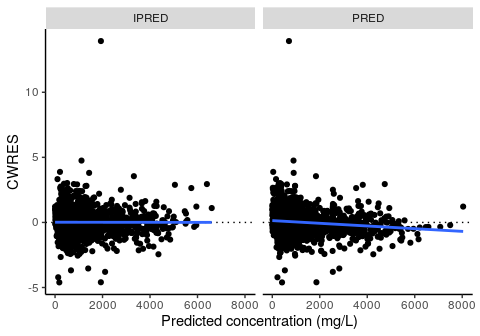


Beside a few outliers, the goodness-of-fit plots show good agreement between the predictions and the observed data.

## Combined model

The basic goodness-of-fit plot for the final joint model showed no bias when plotted versus time or versus predicted concentrations. This was corroborated by the (prediction-corrected) visual predictive checks, which showed that the model predicts the data well for both q6 and q24 dosing, with only minor deviations of predicted from the observed median and percentiles.


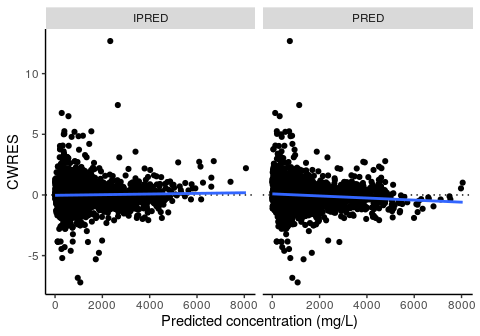


Residual plot versus predicted concentration


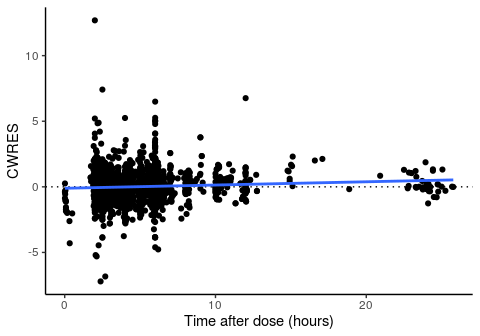


Residual plot versus time after dose

## Visual predictive checks final model


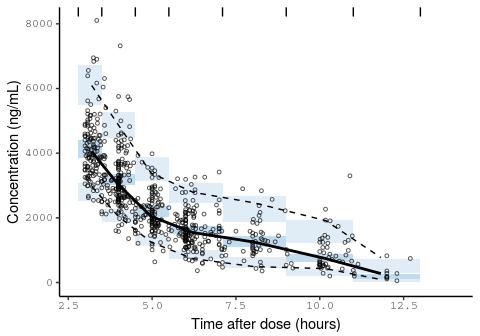


Prediction-corrected VPC for q24 dosing


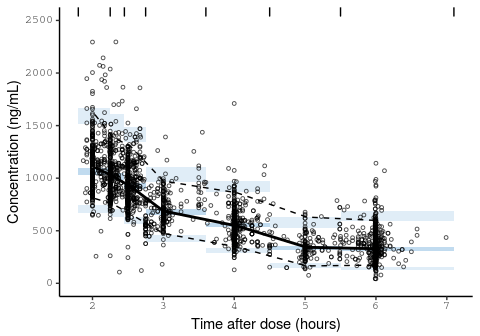


Prediction-corrected VPC for q6 dosing

# Discussion

The final structural model that best defined both populations is a one-compartment model, with covariates age, fat-free-mass, conditioning regimen, and day of treatment on clearance, as well as fat-free-mass on volume of distribution. Parameter estimates are provided in the table below.

The non-linear elimination initially reported in one of the models was removed as it was not supported by the data in neither of the populations studied. It must be noted that the original model that reported non-linear CL was only non-linear to a minor extent, as the KM value was reported to be very high, resulting in mostly linear behavior in the common concentration range, and only minor non-linearity at very high concentrations. Furthermore, the hinge-function describing the relationship between age and CL was replaced with a maturation function (as used in the model for the <12kg population), which improved fit while using the same degrees of freedom, and implementing a more mechanistic approach. Finally, allometric scaling was changed to use fat-free-mass instead of measured weight. This showed a significant and relevant improvement in fit, and also supports a more mechanistic interpretation of the busulfan pharmacokinetics. This should especially improve future predictions in obese children.

Concentrations in the >12kg dataset on a q24 regimen were initially underpredicted by a factor of ~25%. After inclusion of the effect of different conditioning regimens, the underprediction was completely resolved, as shown in the VPCs and goodness-of-fit plots. This also matches clinical observations, where for many recent patients the concentrations following the initial dose were unexpectedly high. Unfortunately, the conditioning regimen was only changed after the institution moved from q6 to q24 dosing, so no data was available for the new conditiong regimen on q6 dosing, although some data was available for both regimens and q24 dosing. The observed effect does however not seem to be caused by a concentration-dependent non-linearity in CL (Michaelis-Menten kinetics) due to the change from q6 to q24-hour dosing. From the current data, the most likely explanation seems that the change in conditioning regimen negatively impacted the drug clearance.

It was observed in a small selection of patients that initial concentrations collected shortly after end of infusion were underpredicted greatly by the model, even when using a 2-compartment model. This observation was also made in McCune et al. [4]: “Regarding the distribution process, a sample drawn exactly at the end of the infusion may be too soon to reflect the distribution process predicted from the model. A subset of the data excluding concentration time points drawn within 5 minutes of the end of the infusion was used with the model developed from all of the data”. The same approach was used in development of the current model, and a small number of samples was removed from the analysis. After removal, a 2-compartment model did not support the data better than a 1-compartment model. For use of this model in model-based precision dosing workflows, the clinical team managing and performing TDM sampling should be made aware that busulfan TDM samples should not be taken too early after the infusion is stopped, as distribution is not fully complete.

# Final structural model

CL = θ_CL_ *
 (MAT+(1-MAT)*(1-EXP(-AGE*HL))) *
 (1+ θ_REGI_*REGIMEN) *
 (1+ θ_DAY1_*DAY1) *
 (FFM/12) ^0.75^V = θ_V_ *
 (FFM/12)

# Parameter estimates

| Population parameter | Estimate | Unit | RSE | 95%CI |
| --- | --- | --- | --- | --- |
| CL | 3.96 | L/hr | 2.4% | 3.772-4.148 |
| V | 10.8 | L | 1.2% | 10.5-11.1 |
| MAT | 0.451 | - | 8.6% | 0.375-0.527 |
| HL | 1.37 | Years | 18.2% | 0.88-1.86 |
| Eff. conditioning regimen ~ CL | -0.2 | - | 25.8% | -0.301--0.099 |
| Eff. treatment day ~ CL | -0.135 | - | 10% | -0.161--0.109 |
| Additive error | 22.2 | ng/mL | 19.9% | 13.6-30.9 |
| proportional error | 10.6 | % | 7.4% | 0.091-0.121 |

| Variability parameter | % | Var. est | SE | RSE | Shrinkage |
| --- | --- | --- | --- | --- | --- |
| Variability in CL | 24% | 0.0595 | 0.0068 | 11.4% | 7.8% |
| Correlation CL~V | 24% | 0.0323 | 0.0053 | 16.5% | . |
| Variability in V | 54% | 0.0293 | 0.0051 | 17.4% | 15.3% |
| IOV in CL | 13% | 0.0166 | 0.0034 | 20.5% | 42.7% |
| IOV in V | 13% | 0.0178 | 0.0058 | 32.7% | 46.2% |

| Error parameters | Estimate | Unit | RSE | 95%CI |
| --- | --- | --- | --- | --- |
| Additive error | 22.2 | ng/mL | 19.9% | 13.5 - 30.9 |
| proportional error | 10.6 | % | 7.4% | 9.1 -12.1 |

#

# References

1. Savic RM, Cowan MJ, Dvorak CC, Pai S-Y, Pereira L, Bartelink IH, et al. Effect of weight and maturation on busulfan clearance in infants and small children undergoing hematopoietic cell transplantation. Biol Blood Marrow Transplant. 2013 Nov;19(11):1608–14.

2. Long-Boyle JR, Savic R, Yan S, Bartelink I, Musick L, French D, et al. Population pharmacokinetics of busulfan in pediatric and young adult patients undergoing hematopoietic cell transplant: A model-based dosing algorithm for personalized therapy and implementation into routine clinical use. Ther Drug Monit. 2015 Apr;37(2):236–45.

3. Al-Sallami HS, Goulding A, Grant A, Taylor R, Holford N, Duffull SB. Prediction of Fat-Free Mass in Children. Clinical Pharmacokinetics [Internet]. 2015 Nov [cited 2018 Nov 21];54(11):1169–78. Available from: <http://link.springer.com/10.1007/s40262-015-0277-z>

4. McCune JS, Bemer MJ, Barrett JS, Scott Baker K, Gamis AS, Holford NHG. Busulfan in infant to adult hematopoietic cell transplant recipients: A population pharmacokinetic model for initial and bayesian dose personalization. Clin Cancer Res. 2014;20(3):754–63.

5. Bartelink IH, Boelens JJ, Bredius RGM, Egberts ACG, Wang C, Bierings MB, et al. Body Weight-Dependent pharmacokinetics of busulfan in paediatric haematopoietic stem cell transplantation patients: Towards individualized dosing. Clinical Pharmacokinetics. 2012;51(5):331–45.
